# Drastic reduction in the intensity of Poaceae pollen season in Central Europe (Lublin, Poland) in 22 years

**DOI:** 10.1371/journal.pone.0336972

**Published:** 2025-11-19

**Authors:** Krystyna Piotrowska-Weryszko, Agnieszka Kubik-Komar, Elżbieta Weryszko-Chmielewska, Agata Konarska, Aneta Sulborska-Różycka

**Affiliations:** 1 Department of Botany and Plant Physiology, University of Life Sciences in Lublin, Lublin, Poland; 2 Department of Applied Mathematics and Computer Science, University of Life Sciences in Lublin, Lublin, Poland; Government College Women University Sialkot: GC Women University Sialkot, PAKISTAN

## Abstract

Grass pollen grains cause allergic reactions in a large portion of the global population. Aerobiological monitoring provides a valuable method for assessing plant responses to climate change. Wind-pollinated plants exhibit varying responses to climate shifts, and this study aimed to analyze long-term trends in Poaceae pollen concentration in Lublin (Poland) from 2001 to 2022. Pollen seasons were analyzed using the volumetric method. The following parameters were assessed for each season: onset, end, duration, peak value, date of peak, number of high-pollen days, and annual pollen sum. Meteorological data were collected for the same period. Statistical analysis was performed using Spearman’s rank correlation to examine relationships between pollen season parameters and meteorological factors, and multivariate regression models were developed to explore the influence of specific climate variables on pollen season dynamics. Additionally, principal component analysis (PCA) was applied for visual comparisons of grass seasons. The analysis revealed a significant decline in the annual pollen sum, the July pollen sum, and the number of high pollen days, with the most substantial changes occurring in July. The decrease in the annual pollen sum was significantly associated with lower humidity in March and May and higher temperatures in June. Peak pollen values were negatively correlated with increased sunshine in March and April and positively correlated with humidity in March and May, while humidity levels strongly influenced the number of high-pollen days. The intensity of the grass pollen season notably weakened after 2011, in alignment with broader trends observed across Europe, attributed to climate change. These findings highlight the substantial influence of climatic variables on the grass pollen season, with implications for individuals with respiratory allergies. The results also underscore the broader public health and ecological impacts of climate change, suggesting the need for continued monitoring and adaptive measures.

## Introduction

### Ecological significance of grasses

Grasses (Poaceae), including cereals, are one of the most ecologically significant plant families, found across all continents and in various climatic zones and habitats. They are highly diverse, with approximately 800 genera and 12,000 species, predominantly perennial (82%), classified into 12 subfamilies [1,2]. Grassland ecosystems, which encompass meadows, steppes, prairies, pampas, savannas, and Arctic meadows, represent the largest biome on Earth, covering approximately 53 million km², or 31–43% of the global land area not covered by permanent ice [3–5]. These ecosystems are characterized by a rich diversity of species, contributing significantly to global biodiversity [6,7].

### Reproductive biology of grasses and pollen characteristics

The reproductive structures of Poaceae species are typically spikes or panicles, containing numerous small flowers arranged in spikelets [8]. As wind-pollinated plants, grasses produce large quantities of dry, lightweight pollen to maximize the chances of successful cross-pollination [9]. The flowers of grass species are small, and the pollen grains are generally spherical or ellipsoidal, with sizes ranging from 16 to 50 µm, though cultivated species like wheat and maize produce larger grains [10,11].

Pollen release in grass species typically occurs over a short period of a few days (e.g., about 5 days for *Secale cereale* and *Poa pratensis*, 6 days for *Lolium perenne*, and 7 days for *Dactylis glomerata*) [12]. These plants are key contributors to the global pollen load, especially during peak pollen seasons.

### Health impacts of grass pollen

Grass pollen is often classified as a major allergen responsible for a large number of allergic reactions worldwide [13]. Pollen allergies affect around 40% of the adult population in Europe and approximately 20% globally, with grass pollen allergies accounting for up to 80% of these cases [13,14]. Common symptoms of grass pollen allergy include seasonal allergic rhinitis, conjunctivitis, and in more severe cases, atopic asthma [15,16]. These allergies cause a substantial public health burden, affecting individuals’ quality of life and leading to increased absenteeism from work and school, as well as greater healthcare costs [17].

In the Northern Hemisphere, the most allergenic grass species belong to three major subfamilies: Pooideae, Chloridoideae, and Panicoideae. Species from these subfamilies, such as *Phleum*, *Dactylis*, *Lolium*, *Trisetum*, and *Poa*, are among the most commonly implicated in allergic reactions [18,19]. The allergenicity of different grass species can vary significantly depending on biological, ecological, and genetic factors, leading to cross-reactions among species, and even between grasses and other plant families (e.g., birch, olive tree, and sunflower) [20,21].

### Climate change and pollen seasons

Climate variables, including temperature, humidity, and sunshine highly influence the timing and intensity of pollen seasons. Climate change has led to shifts in plant phenology, including earlier onset dates and longer pollen seasons for many plant taxa, particularly wind-pollinated species like grasses [22,23]. Higher temperatures have been shown to shorten the duration of the cold period, leading to earlier flowering and, consequently, earlier pollen release [24,25]. Additionally, changes in precipitation patterns and increased frequency of extreme weather events have been linked to altered pollen dynamics [26].

In temperate regions, including Poland, grass pollen seasons typically span from May to October, with peak pollen concentrations occurring in late May to early July. However, the timing of these peaks may vary depending on local climatic conditions. Studies suggest that climate change is extending the duration of pollen seasons in some regions, which may lead to more prolonged and intense allergy symptoms for sensitive individuals [27,28].

### Study rationale and objectives

While there is a growing body of research on the relationship between climate change and pollen seasons, studies focused specifically on Central-Eastern Europe remain limited. This study aims to analyze long-term trends in grass pollen seasons in Lublin, Poland, from 2001 to 2022. We examined how meteorological factors, such as temperature, rainfall, humidity, and sunshine, have influenced the timing, duration, and intensity of the grass pollen season in this region. Given the rising prevalence of pollen allergies and the increasing impact of climate change on plant phenology, this research aims to provide valuable insights for managing pollen exposure and developing strategies to mitigate the health impacts of pollen allergies.

## Materials and methods

### Climate of the study area

Continental air masses primarily influence the climate of the Lublin region. The growing season lasts approximately 215 days. The average annual air temperature, based on data from 1951 to 2000, is 7.4°C, while the average annual precipitation is 550 mm [29].

### Data collection

Airborne pollen monitoring in Lublin (central-eastern Poland) was performed from 2001 to 2022. Daily average concentrations of grass pollen in the air were measured using a Hirst-type pollen trap (Lanzoni VPPS 2000). The device was positioned on the flat roof of a building at the University of Life Sciences in Lublin, 18 meters above ground level (51°14’37“ N, 22°32’25” E; 197 m a.s.l.). The methodology of data collection followed standard methods, in accordance with the recommendations of the European Aerobiology Society [30] and the European Standard [31]. The research methodology was described in detail in the work by Piotrowska-Weryszko et al. [32].

### Grass pollen data

Grass pollen concentrations were expressed as the number of pollen grains per 1 m³ of air per day (P/m³) [33]. The pollen season was determined using the 98% method [34]. The season’s start was defined as the date when 1% of the seasonal cumulative pollen concentration was reached, and the end of the season was defined as when the cumulative pollen sum reached 99% [35].

The following basic parameters of the grass pollen season were analyzed: start, end, duration, maximum pollen concentration (peak value), date of maximum concentration, annual pollen sum, and number of high-pollen days. Additionally, the number of days with threshold concentrations causing allergy symptoms in sensitive individuals (≥20 P/m³) and in all allergic individuals (≥50 P/m³) was calculated [36]. The high-pollen season was defined as beginning on the first day of three consecutive days, each with at least 50 pollen/m³, and ending on the last day of at least three consecutive days, each with at least 50 pollen/m³ [37].

### Statistical analyses

To assess changes over time in all season parameters, linear regression was applied. This widely used technique identifies linear trends and evaluates the strength and direction of temporal changes in the data, with the p-value indicating whether the observed slope is statistically significantly different from zero, and R² expressing the proportion of variance explained by the trend. In addition, normality tests were applied to the residuals of the linear models, with p-values above the significance level indicating an approximately normal distribution.

Spearman’s rank correlation was used to determine relationships between the analyzed season parameters as well as between these parameters and meteorological factors. This method assesses the strength and direction of relationships based on the relative ranking of the data rather than their actual values, making it more robust to outliers and skewed distributions. Associations were considered statistically significant when the p-value was less than 0.05. For comparison, data from the period 2011–2022, characterized by lower pollen concentrations, were also subjected to correlation analysis.

Multiple regression analysis was employed to develop models describing the relationships between season parameters and meteorological factors, using monthly values of average, maximum, and minimum air temperature, cloud cover, sunshine, relative humidity, and rainfall. These models provide insights into how meteorological factors interact to shape the characteristics of the pollen season and could also be applied to predict future pollen seasons. In multiple regression, the p-value for the overall model indicates whether the set of predictors collectively explains a statistically significant portion of the variance in the dependent variable. The fit of the regression models was evaluated using R², and the residuals were checked for normality to verify model assumptions.

Principal Component Analysis (PCA) was performed to reduce the dimensionality of the data by identifying orthogonal variables (principal components) – PC1, PC2, and PC3. By transforming the original variables into a smaller set of uncorrelated components, PCA enhances data visualization and allows for more effective comparison. Plots displaying the distribution of pollen seasons in coordinate systems based on the principal components PC1-PC2 and PC1-PC3 were created. These graphical representations facilitated quick visual comparisons of the grass pollen seasons based on their key characteristics. Before conducting PCA, linear relationships between season parameters were assessed, and data sets with Pearson correlation coefficients exceeding 0.7 were excluded to prevent multicollinearity.

The statistical analyses were performed using Microsoft Excel 2013 [38] for data management and basic calculations, and STATISTICA version 13.1 [39] for more advanced statistical modeling and analysis. A significance level of 0.05 was applied to all statistical tests to determine the threshold for rejecting the null hypothesis.

Meteorological data used in the analysis were sourced from the Institute of Meteorology and Water Management (IMGW).

## Results

### Pollen season duration and variability

Over 22 years of research, the Poaceae pollen season in Lublin began as early as the end of April (2018) and as late as the third decade of May (2005). The difference between the extreme dates was 27 days. On average, the pollen season started on May 16 and ended on September 5 (Table 1). The start of the season was negatively correlated with the duration (r = −0.636, p = 0.01) and positively with the peak value (r = 0.438, p = 0.05). Seasons that started earlier usually lasted longer and were characterized by lower peak values. Correlation analysis showed that the pollen season lasted longer when it ended later (r = 0.558, p = 0.01), and a later end date was associated with a later occurrence of the peak value (r = 0.465, p = 0.05). The end of the season was the most stable parameter (V = 2.6%). The season end date varied by 22 days. The Poaceae pollen season in Lublin lasted an average of almost four months (113 days). The difference between the longest (2014) and shortest (2010) seasons was 36 days. The results indicate that the start of the Poaceae pollen season may differ by almost a month in individual years, while its end is characterized by less variability.

**Table 1:** The statistics of the Poaceae pollen season features in Lublin from data 2001-2022.

Statistics	Pollen season (98%)		Duration	Peak		Pollen
	Start	End	(days)	P/m^3^	Date	sum
Mean	16.05	05.09	113	397	01.07	5755
Min	29.04 *(2018)*	22.08 *(2021)*	92 *(2022)*	156 *(2022)*	10.06 *(2022)*	2596 *(2022)*
Max	26.05 *(2005)*	12.09 *(2004)*	128 *(2002)*	643 *(2008)*	10.07 *(2004)*	8301 *(2010)*
SD	6.4	6.4	8.3	128.7	7.5	1304.5
V (%)	4.7	2.6	7.4	32.5	4.1	22.7

The number of days with grass pollen concentration at which allergy symptoms occur in hypersensitive people (≥20 P/m³) and in all allergic people (≥50 P/m³) was calculated. During the study period, an average of 55 days with pollen concentration values exceeding 20 P/m^3^ per day and 34 days with values exceeding 50 P/m^3^ were recorded. The smallest number of days with a threshold value of 20 P/m^3^ and 50 P/m^3^ was found in 2022, 34 and 20 days, respectively. The highest number of days with a threshold value of 20 P/m^3^ was recorded in 2001 (79 days) and 50 P/m^3^ in 2002 (42 days). The daily pollen concentration at which hypersensitive people experience symptoms (20 P/m^3^) was most often recorded from mid-May, except in 2018 (April 29). These dates coincide with the start of the pollen season, determined by the 98% method. Based on 22 years of daily averages of grass pollen concentrations, it was determined that the average high pollen season with pollen concentrations ≥50 P/m^3^ occurred between 2.06 and 10.07 (38 days), with the beginning occurring between 18.05 and 18.06, and the end between 30.06 and 21.07.

The results indicate that the number and timing of high pollen days vary from year to year. High grass pollen concentrations in Lublin may occur between 18.05 and 21.07.

### Peak values and timing

The maximum daily concentration of grass pollen occurred between June 10 and July 10, on average on July 1 (Table 1). Among the analyzed season characteristics, the peak value showed the highest variability (V = 32.5%). The highest maximum daily pollen concentration was recorded in 2008, over four times higher than in 2022, which had the lowest concentration.

**Fig 1:**
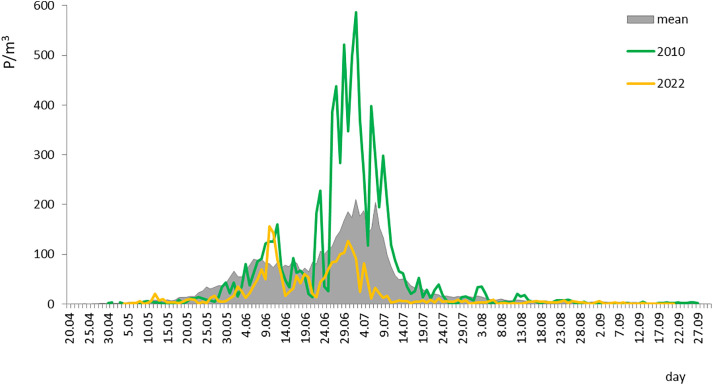
Poaceae daily pollen concentrations in years with the highest (2010) and lowest (2022) annual pollen sum compared with the mean of 22 years.

Pollen seasons typically showed two periods of high concentrations: a smaller peak in early June and a higher peak in early July. The daily pollen concentration dynamics varied between years (Fig 1). The 2022 season stood out, with the lowest peak value and annual sum and the earliest seasonal peak on June 10 (Table 1, Fig 1). The 2004 season, which ended the latest, also had the latest peak date (July 10). The shortest season (2010) was the most abundant, with the highest annual pollen sum (Table 1).

A negative correlation was found between duration and peak value (r = −0.583, p = 0.01), indicating that shorter seasons typically had higher peaks. The peak value was positively correlated with the annual pollen sum (r = 0.623, p = 0.01).

The results suggest that shorter pollen seasons tend to be more intense, with higher daily peak concentrations, and the magnitude of peak pollen levels can vary greatly between years, making the intensity of exposure less predictable than its seasonal occurrence.

### Trends over time

**Fig 2:**
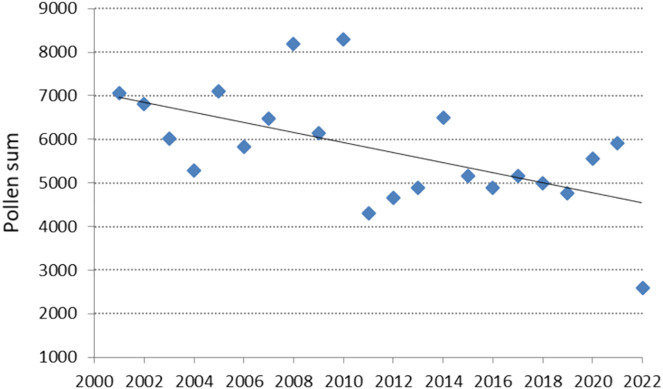
Annual pollen sum of Poaceae in Lublin from 2001 to 2022 and the trend line. $\begin{array}{c} y=-115.7x+\;238480,\;R^{2}=0\mathrm{.33} \end{array}$.

The annual grass pollen sum ranged from 2,596–8,301 grains. The lowest sum was recorded in 2022, and the highest (about four times larger) in 2010. A clear decreasing trend in the annual pollen sum was observed (b = −115.7; p = 0.005), indicating a statistically significant decline in atmospheric pollen concentration over the study years (Fig 2). The assumptions regarding the normal distribution of residuals for the model were met (W = 0.97, p = 0.72).

The significant downward trend in grass pollen concentration in Lublin was also confirmed by comparing pollen sums in the earlier (2001–2010) and later (2011–2022) periods. The mean annual pollen sum in the earlier period was 6,722, while in the later period, it was 4,949, representing a reduction of over 26%.

**Fig 3:**
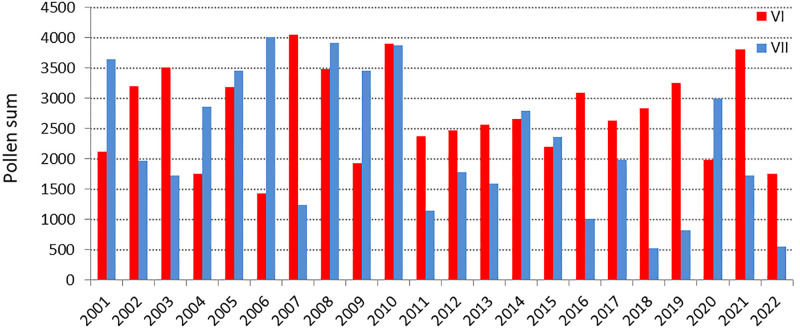
Comparison of Poaceae pollen grain totals in June and July from 2001 to 2022.

A monthly analysis of pollen sums over 22 years showed that the highest concentrations occurred in June and July, with an average of 2,737 and 2,249 grains, respectively. However, in some years, significantly more pollen was recorded in July (Fig 3). In 2018, high average pollen sums were recorded unusually early in April; in June, pollen concentrations were 5.4 times higher than in July.

**Fig 4:**
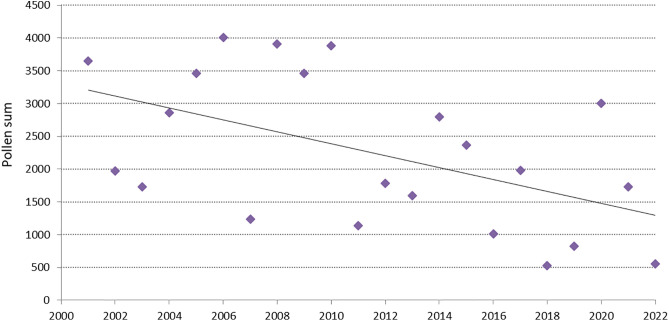
Grass pollen grain totals in July in Lublin from 2001 to 2022 and the trend line. $y\;=\;-\text{91}.\text{005x}\;+\;\text{185306},\;{\text{R}}^{2}\;=\;\text{0}.\text{27}$.

2001−2010 recorded almost 2 times higher average pollen grain totals in July than 2011−2022. A statistically significant decreasing trend (b = −91, p = 0.014) was found for July pollen grain totals during the study years (Fig 4). The assumptions of normal distribution of residuals for this model were met (W = 0.93, p = 0.11). In contrast, no linear trend was observed in June (R^2^ = 0.001), so the decline in pollen concentration over the season occurred mainly in July.

**Fig 5:**
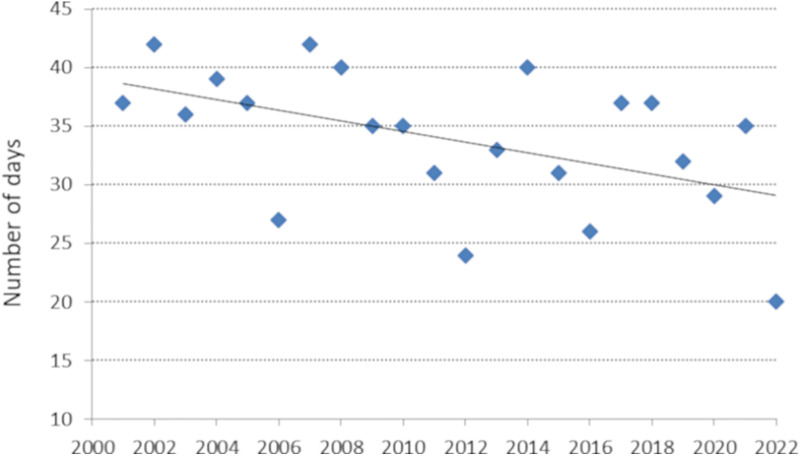
Number of days with daily Poaceae pollen concentration  ≥ 50 grains, or number of high pollen days: y = −0.4512x + 941.37, R² = 0.25.

A statistically significant decreasing trend (b = −0.45, p = 0.02) was observed regarding the number of days with concentrations ≥ 50 grains (number of high pollen days) (Fig 5). The hypothesis of a normal distribution of residuals for this model was satisfied (W = 0.93, p = 0.11).

Over the 22-year study period, a statistically significant downward trend in annual grass pollen sums was observed in Lublin, primarily driven by a marked decline in pollen concentrations during July, which corresponded with a reduction in the number of days exceeding high pollen thresholds. This decline was confirmed by comparing the earlier (2001–2010) and later (2011–2022) decades, indicating substantial changes in seasonal pollen dynamics over time.

### Meteorological influences

One of the most critical factors influencing variability in pollen seasons is weather. Analysis showed that humidity and sunshine in March and temperatures in May and June were key factors controlling the parameters of the grass pollen season in 2001–2022 (Table 2). Peak value was positively correlated with humidity and negatively with sunshine in March. A negative correlation with temperature was found in June for the peak date and annual total sum. This means that the higher temperature in June accelerates the peak date and causes a lower total pollen count. On the other hand, the number of high pollen days depended mainly on humidity in July (positive correlation). Due to the lower pollen concentration in the 2011–2022 period, the relationship between pollen season parameters and weather was also examined. Similar results were obtained for the peak date for both analyzed periods. Higher June temperatures and sunshine duration accelerated the peak value (Table 2). The duration and number of high pollen days in the 2011–2022 period increased with rainfall intensity. These analyses indicate that pollen seasons were less abundant with higher temperatures and sunshine duration, and the peak date occurred earlier. The decrease in peak value was significantly associated with lower air humidity in March.

**Table 2:** Significant Spearman’s correlations between pollen season parameters and meteorological factors in Lublin in the period 2001-2022 and 2011-2022 at the 0.05 significance level.

Season parameter vs. meteorological factor	Spearman’s coefficient	
	2001-2022	2011-2022
**Dependent variable: duration**		
humidity in III	−0.42	–
rainfall in V	–	0.60
**Dependent variable: peak value**		
humidity in III	0.48	–
sunshine in III	−0.44	–
**Dependent variable: peak date**		
sunshine in III	−0.48	–
sunshine in VI	–	−0.61
mean daily average temperature in VI	−0.51	−0.62
mean daily maximum temperature in V	−0.47	–
mean daily maximum temperature in VI	−0.52	−0.68
**Dependent variable: total pollen sum**		
mean daily maximum temperature in VI	−0.50	–
mean daily minimum temperature in VI	−0.49	–
**Dependent variable: number of high pollen days**		
humidity in VII	0.42	–
rainfall in IV	–	0.66

### Predictive modeling

**Fig 6:**
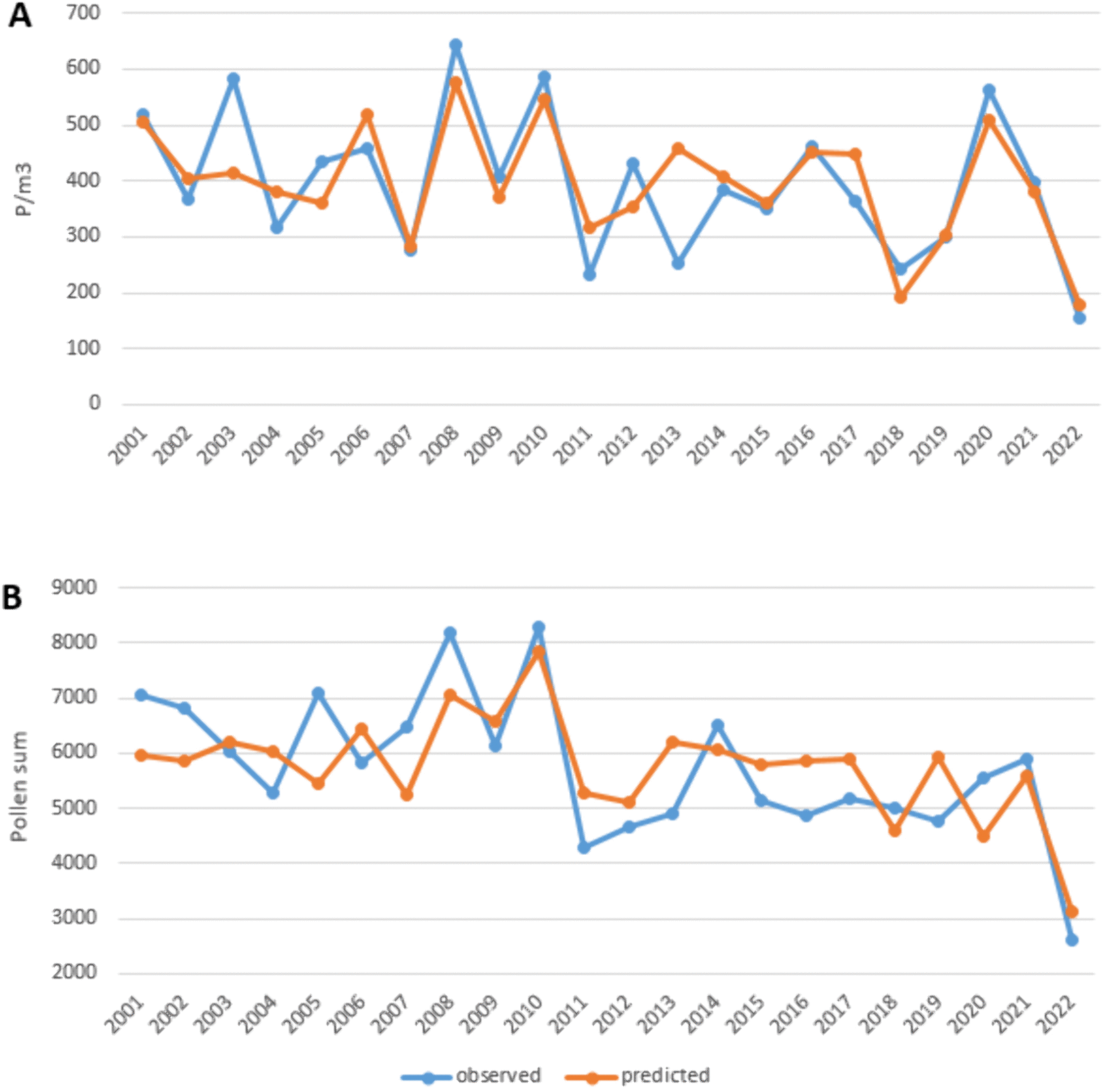
Poaceae peak values. (A) and annual pollen sum (B); observed and predicted values calculated in the regression model.

Predictive models were developed for peak value (1) and annual pollen sum (2) using multiple regression analysis (Fig 6). Results for other season parameters were unsatisfactory and excluded from further analysis. The regression analysis showed that March and May humidity and April sunshine significantly influenced the peak value (F = 11.34, p = 0.0002), while March and May humidity influenced the annual pollen sum (F = 10.41, p = 0.00089). In Fig 6, we can see that for the peak value, the agreement between the value observed in the years 2001–2022 and the one calculated according to the model was relatively high, except for the years 2003 and 2013. A lower agreement was found between the observed value and the one calculated from the model for the annual total. The adjusted R² was 0.6 for the peak value model and 0.47 for the annual sum model. These results indicate that, in addition to the meteorological factors mentioned, there are additional sources of variability of these features that are not captured in the models.

The presented research results from the last two decades indicate that forecasting the course of grass pollen seasons in the central-eastern part of Poland is not compelling enough, which suggests the need for constant monitoring of grass pollen concentration in the air. This action is significant in the treatment of allergies or in avoiding the threat of inhalant allergies. The assumptions regarding the normal distribution of residuals for the models were not rejected (Shapiro-Wilk W = 0.95, p = 0.37; W = 0.93, p = 0.11).

The statistical model describing the relationship between peak value and meteorological conditions is as follows:


$$Peak\;\;value=-\text{1}\text{0}\text{1}\text{3}.\text{1}\text{3}+\text{1}\text{7}.\text{4}\text{6}\cdot {Hum}_{III}-\text{1}.\text{3}\text{8}\cdot {Sun}_{IV}+\text{0}.\text{0}\text{6}\cdot {\left({Hum}_{V}\right)}^{\text{2}}$$ (1)

where:

*Hum*_*II*I_ – humidity in March,

*Sun*_*IV*_ – sunshine in April,

*Hum*_*V*_ – humidity in May.

The regression equation for the annual pollen sum is:


$$Annual\;pollen\;sum\;=-\text{1}\text{0}\text{7}\text{7}\text{4}+\text{1}\text{5}\text{0}.\text{7}\cdot {Hum}_{III}+\text{0}.\text{0}\text{9}\cdot \;{{Hum}_{V}}^{\text{2}}$$ (2)

where:

*Hum*_*II*I_ – humidity in March,

*Hum*_V_ – humidity in May.

The developed models demonstrated that March and May humidity, along with April sunshine, significantly influence the peak grass pollen concentration and annual pollen sum. Still, moderate predictive power and unexplained variability highlight the ongoing need for continuous pollen monitoring to manage allergy risks effectively.

### Principal component analysis

Before the PCA analysis, the linear relationship between the parameters was checked using Pearson’s linear correlation coefficient, and the peak value, which was correlated with the annual pollen sum at 0.71, was excluded from this analysis. In the remaining cases, the absolute value of Pearson’s correlation coefficient was below 0.66.

Results of PCA with VARIMAX rotation identified three principal components explaining over 94% of the total variance. PC1 was related to the start date and negatively to duration (factor loadings: 0.98, −0.74 respectively), PC2 was associated with the season end (factor loading 0.98), and PC3 with peak date and annual pollen sum (factor loadings:0.82, 0.84 respectively).

**Fig 7:**
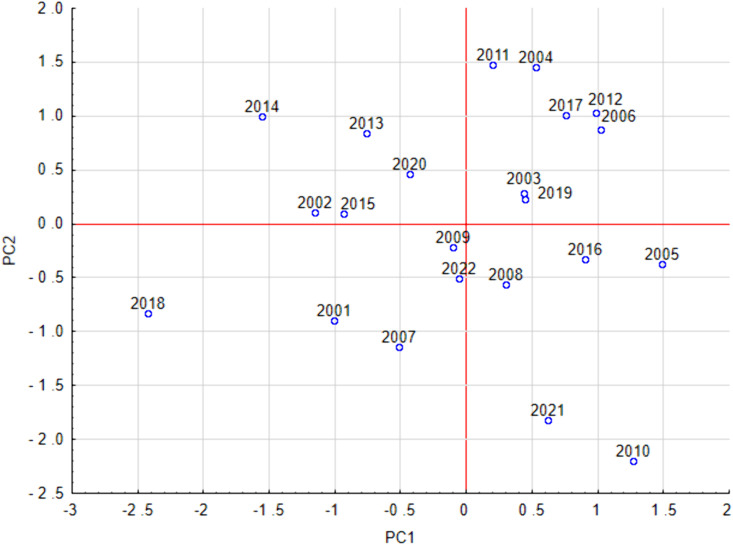
Factor scores in the PC1-PC2 coordinate system. PC1 positively correlated with Start and negatively correlated with Duration, and PC2 positively correlated with End.

The PC1–PC2 plot analysis showed that the 2018 season was relatively long, with an early start, while 2005 and 2010 were short, with late starts. The 2010 season, like 2021, ended quickly, in contrast to the seasons we can see at the top of the graph – 2004, 2012, 2017, 2006, 2014, and 2013 (Fig 7). Statistical analyses show that early grass pollen seasons occurred in similar numbers in both the earlier (2001–2010) and later (2011–2022) study periods meaning that there were no significant changes in the start dates and length of the grass pollen season (Fig 7).

**Fig 8:**
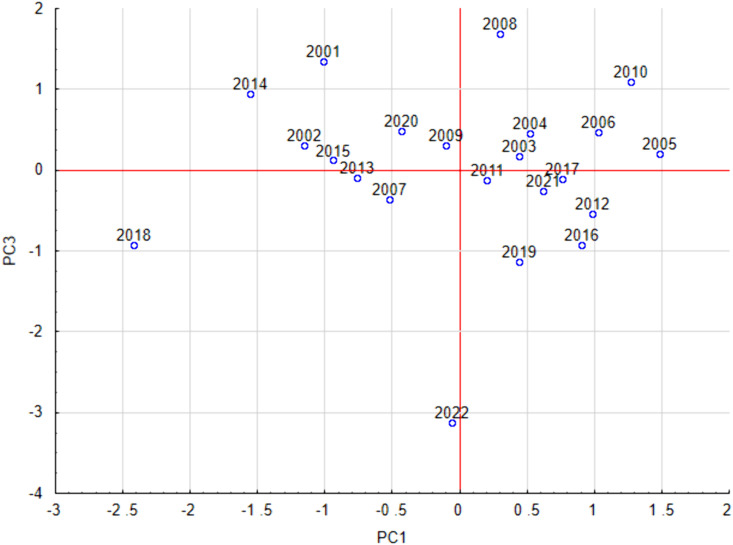
Factor scores in the PC1-PC3 coordinate system. PC1 positively correlated with Start and negatively correlated with Duration, and PC3 positively correlated with Peak Date and Annual total.

Analysis of the PC1-PC3 graph also identifies more abundant seasons with relatively late peak seasons, such as 2008, 2010, 2001, and 2014, as well as less abundant seasons. In addition, a clear outlier can be seen in 2022, characterized by an early peak and low pollen abundance in the air (Fig 8). Statistical analysis confirmed our observations about the different intensity of pollen seasons in the earlier and later study periods. It turned out that among the pollen seasons of the 2001–2010 period, 90% were abundant seasons, while in the 2011–2022 period, 75% were less abundant seasons. In the later study period, the abundance of pollen seasons decreased significantly (Fig 8).

Principal Component Analysis identified three main components explaining over 94% of the variance in grass pollen season parameters, corresponding to season start and duration, season end, and peak date with pollen abundance. The distribution of seasons in the PC1–PC2 and PC1–PC3 plots revealed considerable variability in season length, timing, and intensity across years. Notably, pollen abundance showed an evident decline in the later study period (2011–2022) compared to the earlier period (2001–2010). The 2022 season stood out with an unusually early peak and markedly low pollen concentrations.

## Discussion

### Intensity of pollen season in Lublin

Lublin, located in the central-eastern region of Poland, has so far recorded the highest grass pollen concentrations in the air in the country, as shown by the results of previous work. Comparative research conducted in eight Polish cities between 2001 and 2014 showed that Lublin’s average annual grass pollen sum was the highest, amounting to 5965 grains [40]. These findings are further supported by data from 2015 [41], 2018, and 2019 [42,43], where annual grass pollen sums for Lublin were again the highest at 5149, 4973, and 4715 grains, respectively. In other cities during the same years, these values ranged from 3308–4492 in 2015 [41], 1593–4086 in 2018 [42], and 1256–4106 in 2019 [43].

### Temporal trends and shifts

In Lublin, the onset of the Poaceae pollen season showed temporal variability, with slight advancement and no significant trend. Additionally, no significant trend was observed for the duration of the pollen season. However, we identified a significant decreasing trend in the duration of the high pollen season. Humidity in March proved to be an important factor influencing season duration. Lublin’s mean annual grass pollen sum over the 22 years of research (2001–2022) was 5755 grains. This period exhibited significant variability, ranging from 2596 to 8301 grains. Trend analysis revealed a strong, statistically significant downward trend for the annual pollen sum in Lublin from 2001–2022. Based on the trend line, the decline exceeded 34%. A notable decrease (approximately 30%) in peak values was also observed, though this reduction was not statistically significant.

Strong evidence of this downward trend is further provided by comparing annual pollen sums from the two study periods: 6722 grains in 2001–2010 and 4949 grains in 2011–2022, representing a decline of over 26%. Since 2011, we have observed a decrease in annual grass pollen totals in Lublin, as confirmed by statistical analysis. Research from 2001–2012 indicated that the trend line for annual grass pollen totals in Lublin was nearly flat [44], suggesting that more substantial changes occurred in the later part of the study period. The representation of grass pollen in the pollen spectrum in this city in 2001–2010 was 9.98%, while in 2011–2022, it was reduced to 7.25%. It is essential to highlight that during these two periods (10 and 12 years), the total average annual pollen sums for all taxa were similar, at 69596 and 70240 grains, respectively. These data clearly indicate a drastic reduction in the severity of grass pollen seasons in Lublin from 2001–2022, with no corresponding change in the total pollen load from all taxa.

### Meteorological and climate influences

Our research shows that annual grass pollen sums were significantly influenced by humidity in March and May and temperatures in June, while peak values were additionally affected by sunshine in March and April. These weather variables are associated with climate change, characterized by more extreme temperature and rainfall events [45]. Research by Sachindra et al. [46] in Lublin clearly indicates climate warming over the past 20 years. The authors demonstrated that maximum summer temperatures between 1998–2020 increased by 0.083°C/year compared to 1974–1997, amounting to a 1.9°C increase over 23 years. These results seem to explain the significant decline in grass pollen season intensity in July, as demonstrated in our study.

The decrease in the intensity of grass pollen seasons in the Lublin region, apart from the increased temperature, could also have been influenced by the lower amount of precipitation and reduced humidity associated with climate change. Kołodziej et al. [47] showed that in the Lublin area, rainfall deficits occurred already in the years 1951–2000 in June and July. Moreover, in Poland, an increase in the dry areas by 30% was noted in the years 1981–2010, while in the years 1971–2000 it was only 5% [48]. The results of statistical analysis indicate that, in addition to meteorological factors, there are other sources of variability in pollen season characteristics. These may include grassland being replaced by urban areas and land-use changes.

### Comparisons with other cities and regions

An earlier start of the grass pollen season has been observed in other Polish research centers (e.g., Kraków) [49]. In Poznań, an increase in pollen season duration was reported, positively correlated with rising summer temperatures [50]. The relative stability in the length of grass pollen seasons in eight Polish cities was reported by [40].

Elsewhere in Europe, results concerning these seasonal characteristics vary. In Denmark, phenological data from the past 40 years indicated earlier and longer grass pollen seasons, with maximum pollen loads occurring earlier [51]. Similarly, in Évora City (Portugal), Poaceae pollen seasons were found to last longer [52]. Consistent with our findings, no changes in the length of grass pollen seasons were observed in several UK cities [53].

A slight decreasing trend in Poaceae pollen season intensity was also observed in Poznań from 1996 to 2011 [50]. Reductions in the severity of grass pollen seasons have also been recorded in several European countries. In northern Italy (Parma), the Seasonal Pollen Index (SPI) of Poaceae significantly decreased between 1994 and 2011, which the authors attribute to climate change, environmental change, and land use changes [54]. Similar trends have been observed in Slovakia [55] and in Belgium, the Netherlands, and Luxembourg, where Poaceae annual pollen integral and peak values showed a declining trend [56,57]. In Switzerland, a decreasing trend in Poaceae SPI was recorded at three of six measurement sites [58]. Conversely, a flat trend in Poaceae SPI was observed in Sweden [59], Switzerland [60], and the UK [53]. In Oklahoma (USA), Levetin [61] also recorded a reduction in Poaceae pollen season intensity. However, the opposite trend was observed in Portugal over 21 years, where an increase in annual pollen totals and the number of high pollen days was documented [52]. A slight upward trend in Poaceae pollen load has been reported in Northern Italy [62].

Our research, along with the findings of other cited authors, indicates that climate change has altered the intensity of grass pollen loads across Europe. In Central Europe (Poland, Slovakia) and Western Europe (Belgium, the Netherlands, Luxembourg, Switzerland), concentrations of grass pollen have declined to varying degrees. In contrast, Southern Europe (Portugal, Italy) has shown an upward trend, while Northern European countries (Sweden, UK) display relatively stable levels. These patterns are likely linked to the geographic location of study sites, their climatic zones, and vegetation types.

### Concluding insights

Allergy symptoms manifest with the appearance of pollen grains in the air, so the differences observed in the start of pollen seasons are important for allergic individuals. The start of the grass pollen season can vary by almost a month from year to year, making it difficult to predict. Therefore, allergy sufferers need to maintain constant pollen monitoring and follow alerts.

In studies conducted in Lublin between 2001 and 2022, we observed a statistically significant downward trend in annual grass pollen counts, as well as a decrease in pollen grain counts in July, and a reduction in the number of days with high pollen concentrations. The lower abundance of grass pollen seasons is associated with lower exposure to allergenic pollen grains, which may contribute to less severe symptoms in individuals with pollen allergies or reduced allergy cases.

Multiple studies confirm that climate change affects public health through alterations in pollen release [63]. Our long-term research, together with studies conducted in Central and Western Europe, points to a decline in seasonal Poaceae pollen loads. This reduction may lower the risk for sensitized individuals, potentially alleviating allergic rhinitis and asthma symptoms. By contrast, the increase observed in Southern Europe suggests a heightened risk, likely leading to more frequent respiratory issues among allergy sufferers.

The marked decline in grass pollen production and the shortening of peak pollen seasons represent strong ecological signals. These trends may reduce pollination success and seed output [64,65], disrupt food chains [66], limit gene flow between populations, and weaken adaptive capacity to climate change [67]. They may also alter the structure and functioning of ecosystems [65], ultimately driving permanent biodiversity changes. Such shifts are valuable bioindicators of climatic stress and habitat degradation.

Current climate change, as confirmed in our study, is characterized mainly by rising temperatures. Elevated temperatures during flower development have been shown to disrupt pollen formation, reducing both the quantity and quality of grains. Under these conditions, pollen viability, germination, and tube growth can all be impaired [68]. In addition, reduced soil water availability under changing climatic conditions may accelerate, delay, or inhibit flowering in Poaceae, depending on the timing and severity of stress [69]. Drought constrains photosynthesis, which in turn reduces the number of inflorescences and seed size [70]. Water stress during flower formation can even result in flower abortion [71].

Air humidity is also a crucial factor, influencing anther dehiscence and pollen release. Excessively low humidity accelerates pollen dehydration and decreases viability [72]. In dry habitats, flowers often have shorter lifespans and produce fewer viable pollen grains [73]. Aerobiological studies documenting lower grass pollen levels under certain environmental conditions underscore the importance of humidity in pollen production.

Land-use change represents another contributing factor. Recent reductions in cultivated and natural vegetation [74] are driven mainly by industrial expansion, transport infrastructure, and urbanization [75]. These pressures not only reduce pollen production but also introduce additional stressors, such as higher temperatures, air pollutants, and habitat fragmentation. Such processes can simultaneously lower pollen outputs and increase the diversity of allergenic plants.

Despite their allergenic properties, grasses should remain integral to urban planning, as they provide essential ecological services. Risks can be mitigated by mowing before flowering. For individuals allergic to grass pollen, monitoring regional pollen forecasts and weather updates remains crucial. The observed decline in grass pollen intensity in Central and Western Europe may signal a reduced allergenic burden in these regions.

Grasses also have potential in sustainable urban design, contributing to cooling, stormwater management, and pollution control. Innovative green infrastructure – such as parks, squares, grass-covered streets, green roofs, living walls, and rain gardens – can integrate grasses to enhance biodiversity and improve environmental resilience.

## Conclusions

- The results of our study reveal significant changes in the timing and intensity of grass pollen seasons in central-eastern Poland over the past 22 years. These findings are crucial for researchers studying environmental variations and may have substantial implications for individuals dealing with pollinosis.
- Grass pollen production in this region of Poland has decreased notably during the study period, as evidenced by a 34% reduction in the annual pollen sum and a decline in the number of high-pollen days.
- The observed trends are caused by climatic factors, including the negative impact of increasing temperature and sunshine, as well as reduced humidity. Additionally, land-use changes – such as replacing grasslands with urban areas – may have contributed to these shifts.
- The results obtained in Lublin regarding the reduction in the intensity of grass pollen seasons are consistent with general trends observed in Central and Western Europe.
- The highest concentrations of grass pollen in Lublin were recorded in June and July. While we observed a significant decrease in pollen intensity in July, the pollen load in June remained relatively unchanged.
- Our analysis shows that the reduction in grass pollen intensity was most pronounced in the later years of the study, particularly between 2011 and 2022.
- The decrease in pollen intensity did not coincide with significant changes in the pollen season’s start, end, or duration. Our research results suggest that while Poaceae pollen levels decreased, the seasonal window for the presence of other pollen grains remained stable.
- The decline in the intensity of grass pollen seasons, which is occurring in Central Europe, may be positive news for allergy sufferers, as it is associated with a lower risk of respiratory symptoms.
- The demonstrated decline in the quantity of grass pollen grains may imply some negative ecological changes, including reduced plant adaptability, disruptions in food chains, and reduced biodiversity.

## Supporting information

S1 Table The values of the atmospheric pollen parameters. (XLSX)
